# Reduced *in vitro *susceptibility to artemisinin derivatives associated with multi-resistance in a traveller returning from South-East Asia

**DOI:** 10.1186/1475-2875-10-268

**Published:** 2011-09-18

**Authors:** Bruno Pradines, Lionel Bertaux, Christelle Pomares, Pascal Delaunay, Pierre Marty

**Affiliations:** 1 Unité de Recherche en Biologie et Epidémiologie Parasitaires - Unité de Recherche pour les Maladies Infectieuses et Tropicales Emergentes - UMR 6236, Institut de Médecine Tropicale du Service de Santé des Armées, Marseille, France; 2 Centre National de Référence du Paludisme, Marseille, France; 3 Unité de Recherche en Physiologie et Pharmacocinétique Parasitaires - UMR-MD3 Relations Hôte-Parasites - Pharmacologie et Thérapeutique, Institut de Médecine Tropicale du Service de Santé des Armées, Marseille, France; 4 Laboratoire de Parasitologie-Mycologie, Centre Hospitalier Universitaire l'Archet, Nice, France; 5 Inserm U 895, Equipe 6, Université de Nice Sophia Antipolis, Nice, France

## Abstract

Decreased *in vitro *susceptibility to dihydroartemisinin (21.2 nM) and artesunate (16.3 nM) associated with decreased susceptibility or resistance to quinine (1131 nM), mefloquine (166 nM), lumefantrine (114 nM), pyronaridine (70.5 nM) and piperaquine (91.1 nM) is reported in a patient returning from South-East Asia after trekking along the Mekong from the south of Laos to the north of Thailand. Decreased *in vitro *susceptibility to artemisinin derivatives did not appear to be mediated by the number of copies of *pfmdr1 *or *pfATPase6, pfcrt, pfmdr1 *or *pfmrp *polymorphism. The high IC_50 _to mefloquine of this Asian isolate was not associated with *pfmdr1 *copy number. *Pfnhe-1 *microsatellite ms4760 showed a profile 7 (ms4760-7) with three repeats of DNNND and one repeat of DDDNHNDNHNN, which is associated with high quinine reduced susceptibility. The patient recovered in three days without relapse after treatment with the association of quinine and doxycycline. Decreased *in vitro *susceptibility to quinine and the delayed effect of doxycycline may both have contributed to the delayed parasite clearance time, D4 (0.5%) and D7 (0.004%). The *in vitro *data, with IC_50 _for dihydroartemisinin and artesunate were up to ten times those of the reference clone W2, which suggests that this isolate may be resistant to artemisinin derivatives, associated with a decreased susceptibility to quinine.

## Background

Artemisinin-based combination therapy (ACT) is now recommended by the World Health Organization as first-line treatment of uncomplicated falciparum malaria in all areas in which malaria is endemic. However, recent reports from Cambodia of delayed parasite clearance after treatment by ACT have now been confirmed [1]. The resistant phenotype is not yet reflected by the results of conventional *in vitro *drug susceptibility assays. Parasites with slow clearance rate after ACT did not show *in vitro *decreased susceptibility [1]. *In vitro *decreased susceptibility to artemisinin derivatives was never or very rarely reported in Cambodia [1-4]. No molecular marker has been identified, which impedes surveillance studies to monitor the spread of artemisinin resistant phenotype. Decrease of *in vitro *susceptibility to dihydroartemisinin and artesunate, associated with reduced susceptibility to standard anti-malarials, such as quinine, mefloquine and lumefantrine, and new drugs, such as pyronaridine and piperaquine, is reported here.

## Case presentation

A 52-year old female visited rural areas in Laos (Nov 9 to 12, 2009), Cambodia (Nov 12 to 29) and Thailand (Nov 29 to Dec 1). She took part in trekking along the Mekong from the south of Laos to the north of Thailand. The patient presented with fever since November 29 and was hospitalized (Dec 2) in intensive care unit (Centre Hospitalier Universitaire l'Archet, Nice, France) for complicated malaria (15% parasitaemia and altered consciousness). The patient used irregularly doxycycline (100 mg/day) as chemoprophylaxis. The patient was treated by intra-venous quinine chlorhydrate (25 mg/kg/day) and doxycycline (200 mg/day) for seven days. The patient recovered in three days without relapse and was discharged.

*Plasmodium falciparum *parasites were identified at Day 0 (15%), D4 (0.5%) and D7 (0.004%) but were not detected at D43.

## Methods

*In vitro *testing of drug susceptibility was performed by the standard 42-hour ^3^H-hypoxanthine uptake inhibition method [5]. Susceptibility to dihydroartemisinin, artesunate, and ten standard or new anti-malarial drugs, ie chloroquine, quinine, mefloquine, lumefantrine, monodesethylamodiaquine (biologically active metabolite of amodiaquine), pyronaridine, piperaquine, atovaquone, doxycycline and pyrimethamine, was assessed. The laboratory-adapted clone W2, tested on the same day, was used as a reference. Isolates from imported malaria, tested on the same batch of plates, were used as comparators.

Polymorphisms of *pfcrt, pfmdr1, pfmrp *and *pfnhe-1*, involved in quinoline resistance, and in *pfATPase6*, postulated to be involved in artemisinin resistance, and the copy number of *pfmdr1 *were assessed [6].

The French malaria consensus [7] and the WHO [8] recommend to clinically examine patient and control parasitaemia at D0, D3, D7 and D28 to evaluate anti-malarial efficacy. Blood controls were performed at D0, D4, D7 and D43. The genotyping of parasites was assessed at D0, D4 and D7 using six microsatellite loci (microsatellites 7A11, pf2689, pf2802, C4M79, TRAP, C4M69) [9], *msp1 *and *msp2 *[10].

## Consent

Informed consent was not required as the sampling procedures and testing are part of the French national recommendations for the care and surveillance of malaria.

## Results

This isolate showed decreased susceptibility to dihydroartemisinin (21.2 nM) and artesunate (16.3 nM) associated with decreased susceptibility or resistance to quinine (1131 nM), mefloquine (166 nM), lumefantrine (114 nM), pyronaridine (70.5 nM) and piperaquine (91.1 nM) with high ratio in comparison with W2 (Table 1). These IC_50 _and W2 ratios were higher than those of other imported isolates.

**Table 1:** *In vitro *susceptibility to standard antimalarial drugs of the multidrug-resistant isolate in comparison with *P.falciparum *W2 clone and *P. falciparum *isolates tested with the same plate batches

Drugs	Isolate IC_50_	Ratio IC_50 _Isolate/W2	W2* IC_50_	Isolates** Mean IC_50 _(CI95%)	Ratio IC_50 _Isolates/W2	Resistance cut-off
Dihydroartemisinin	21.2 nM	11.8	1.8 nM	2.2 nM (1.3-3.7)	1.2	> 10.5 nM
Artesunate	16.3 nM	10.2	1.6 nM	1.9 nM (1.0-3.2)	1.2	> 10.5 nM
Quinine	1131 nM	1.5	731 nM	201 nM (131-307)	0.3	> 800 nM
Mefloquine	166 nM	4.5	36.6 nM	26.0 nM (15.9-42.5)	0.7	> 30 nM
Lumefantrine	114 nM	4.0	28.4 nM	23.9 nM (14.1-40.8)	0.8	> 150 nM
Pyronaridine	70.5 nM	7.8	9.0 nM	26.2 nM (15.4-44.6)	2.9	ND
Piperaquine	91.1 nM	3.3	27.3 nM	66.2 nM (34.7-126.3)	2.4	ND
Chloroquine	63 nM	0.14	449 nM	63 nM (29-138)	0.1	> 100 nM
Monodesethylamodiaquine	34.4 nM	0.54	63.2 nM	32.4 nM (19.2-54.8)	0.5	> 80 nM
Atovaquone	2.21 nM	0.74	2.99 nM	1.48 nM (1.06-2.07)	0.5	> 490 nM
Doxycycline	18.5 μM	1.5	12.1 μM	10.0 μM (7.8-12.9)	0.8	> 35 μM
Pyrimethamine	497 nM	0.04	12621 nM	107 nM (7-1681)	0.01	> 2000 nM

* The laboratory-adapted clone W2, tested on the same day, was used as a reference.

** Values are geometric mean and 95% confidence interval of 16 isolates from imported malaria, tested on the same batch of plates, and used as comparators.

ND: not determined

Mutations were not identified in *pfmdr1, pfcrt *and *pfmrp *genes. Only one copy of *pfmdr1 *was found. Two synonymous mutations were detected in *pfATPase6 *(N460N and I898I), which were previously described [11]. *Pfnhe-1 *microsatellite ms4760 showed a profile 7 (ms4760-7) with three repeats of DNNND and one repeat of DDDNHNDNHNN, which is associated with high quinine reduced susceptibility [12].

Parasitaemia was controlled at Day 0, D4, D7 and D43. Monoclonal and identical parasites were identified at D0 (15% of parasitaemia), D4 (0.5%) and D7 (0.004%) by using microsatellites 7A11, pf2689, pf2802, C4M79, TRAP, C4M69 and *msp1 *and *msp2*. No parasite was detected at D43.

## Conclusion

This isolate showed reduced susceptibility to artemisinin derivatives, but also to other ACT components commonly used in Asia or in clinical trials, such as mefloquine, lumefantrine, pyronaridine or piperaquine. Surprising, this isolate was susceptible *in vitro *to chloroquine and monodesethylamodiaquine. It was also susceptible to doxycycline. *Plasmodium falciparum *parasite with high IC_50 _to artemisinin and artemether (20.1 nM and 21.4 nM, respectively), but with low IC_50 _to dihydroartemisinin and artesunate (1.8 nM and 6.2 nM, respectively) was recently isolated in a traveller returning from Nigeria, who took artesunate prophylactically (two 50 mg tablets weekly for 4 weeks) [13]. Yet, recent clinical trials of oral artesunate monotherapy suggest that the loss of ACT efficacy might result from decreased efficacy of artemisinin derivatives [1,3]. The median parasite clearance time was 36 hours longer in patients from Western Cambodia, where the efficacy of ACT is decreasing [1]. Nevertheless, this phenomenon was not correlated with artemisinin derivatives IC_50_.

Only one copy of *pfmdr1 *was found. The high IC_50 _to mefloquine of this Asian isolate was not associated with *pfmdr1 *copy number. *Pfnhe-1 *microsatellite ms4760 showed a profile 7 (ms4760-7) with three repeats of DNNND and one repeat of DDDNHNDNHNN, which is associated with high quinine reduced susceptibility [12]. Two repeats of DNNND were seen to be associated with high IC_50 _in quinine clinical failure in traveller from Senegal [14].

The persistence of parasites seven days after the start of treatment with quinine chlorhydrate and doxycycline, but without clinical signs, is consistent with the high IC_50 _and with the profile ms4760 of *pfnhe-1*. The action of doxycycline is delayed. There is a relationship between the amount and duration of exposure and the effect of doxycycline on the erythrocytic stages with an increased activity during the second cycle. Doxycycline has been shown to exert its effect during the first 48 h, but is only detectable in second-generation parasites near the 96 hour time point [15-17]. The French malaria consensus recommends quinine associated with doxycycline for Asian and South-American *P. falciparum *(and not only quinine as for African parasites). Clinical failure with quinine has been shown in a patient returning from French Guiana treated by quinine only [18]. The patient recovered in three days without relapse and left intensive care due to the treatment she received, the association of quinine and doxycycline, as recommended by the French malaria consensus. *In vitro *quinine IC_50 _and *pfnhe-1 *ms4760 analysis suggest a reduced susceptibility to quinine. This decreased susceptibility to quinine and the delayed effect of doxycycline may both have contributed to the delayed parasite clearance time, D4 (0.5%) and D7 (0.004%).

Decreased *in vitro *susceptibility to artemisinin derivatives did not appear to be mediated by the number of copies of *pfmdr1 *or *pfATPase6 *polymorphism. The *in vitro *data, with IC_50 _for dihydroartemisinin and artesunate up to ten times those of the reference clone W2 or the geometric mean of the other isolates, suggest that this isolate could be resistant to artemisinin derivatives, even if there is no evidence that this isolate was clinically resistant to ACT, associated with decreased susceptibility to quinine. And its association with the other in vitro decreased susceptibilities is alarming, especially with the components of ACT used in Asia.

## Competing interests

The authors declare that they have no competing interests.

## Authors' contributions

BP carried out *in vitro *testing of drug susceptibility and drafted the manuscript. LB carried out the molecular genetic studies. CP, PD and PM carried out diagnostic, monitoring of the patient, collection of clinical and epidemiological data and drafted the manuscript. All authors read and approved the final manuscript.
